# Costs and cost-effectiveness of delivering intermittent preventive treatment through schools in western Kenya

**DOI:** 10.1186/1475-2875-7-196

**Published:** 2008-09-30

**Authors:** Matilda Temperley, Dirk H Mueller, J Kiambo Njagi, Willis Akhwale, Siân E Clarke, Matthew CH Jukes, Benson BA Estambale, Simon Brooker

**Affiliations:** 1 London School of Hygiene and Tropical Medicine, Keppel Street, London, WC1E 7HT, UK; 2 Division of Malaria Control, Ministry of Health, Nairobi, Kenya; 3 Harvard Graduate School of Education, Harvard University, Cambridge, MA, USA; 4 Institute of Tropical and Infectious Diseases, University of Nairobi, Nairobi, Kenya; 5 Malaria Public Health and Epidemiology Group, KEMRI/Wellcome Trust Collaborative Programme, Nairobi, Kenya

## Abstract

**Background:**

Awareness of the potential impact of malaria among school-age children has stimulated investigation into malaria interventions that can be delivered through schools. However, little evidence is available on the costs and cost-effectiveness of intervention options. This paper evaluates the costs and cost-effectiveness of intermittent preventive treatment (IPT) as delivered by teachers in schools in western Kenya.

**Methods:**

Information on actual drug and non-drug associated costs were collected from expenditure and salary records, government budgets and interviews with key district and national officials. Effectiveness data were derived from a cluster-randomised-controlled trial of IPT where a single dose of sulphadoxine-pyrimethamine and three daily doses of amodiaquine were provided three times in year (once termly). Both financial and economic costs were estimated from a provider perspective, and effectiveness was estimated in terms of anaemia cases averted. A sensitivity analysis was conducted to assess the impact of key assumptions on estimated cost-effectiveness.

**Results:**

The delivery of IPT by teachers was estimated to cost US$ 1.88 per child treated per year, with drug and teacher training costs constituting the largest cost components. Set-up costs accounted for 13.2% of overall costs (equivalent to US$ 0.25 per child) whilst recurrent costs accounted for 86.8% (US$ 1.63 per child per year). The estimated cost per anaemia case averted was US$ 29.84 and the cost per case of *Plasmodium falciparum *parasitaemia averted was US$ 5.36, respectively. The cost per case of anaemia averted ranged between US$ 24.60 and 40.32 when the prices of antimalarial drugs and delivery costs were varied. Cost-effectiveness was most influenced by effectiveness of IPT and the background prevalence of anaemia. In settings where 30% and 50% of schoolchildren were anaemic, cost-effectiveness ratios were US$ 12.53 and 7.52, respectively.

**Conclusion:**

This study provides the first evidence that IPT administered by teachers is a cost-effective school-based malaria intervention and merits investigation in other settings.

## Introduction

In Africa, there is increasing evidence of the dramatic reductions in malaria mortality and morbidity in early childhood due to recent up-scaling of malaria control efforts [1-4]. There is however some concern that these gains in early childhood may, as a consequence of decreased transmission and a slower acquisition of exposure-dependent immunity, lead to an increased incidence of malaria among older children [5]. Coincidental with this changing epidemiology of malaria, there has been increased recognition of the consequences of malaria in children of school-age, including detrimental effects on haemoglobin levels [6,7] and learning and educational achievement [8,9]. Consequently, there has been a renewed interest in the control of malaria in older children who attend school [10-12]. However, there is currently little international consensus as to the optimal intervention approach. There is also a lack of evidence on the costs and cost-effectiveness of available options for school-based malaria control.

An initial crude cost analysis of options for malaria control in Kenyan schools in 2000 concluded that chemoprophylaxis using the then recommended drug (Proguanil) delivered through schools would be prohibitively expensive [11]. Instead it was suggested that the promotion of prompt and effective diagnosis and treatment in schools would represent an affordable approach to address malaria in schools. However, the practicality and effectiveness of such an approach has only been explored in pilot projects [13-15], and there remain a number of operational challenges in the provision of treatment in schools, including the reliability of diagnosis by non-health personnel, the long term motivation of teachers to play a health role, and challenges associated with the recent introduction of artemisinin combination therapies.

An alternative school-based strategy, already proven effective for protecting pregnant women and infants from malaria-related morbidity, is intermittent preventive treatment (IPT). A recent proof-of-principle trial in western Kenya showed that mass administration of a full therapeutic course of anti-malarial drugs to schoolchildren once a term, irrespective of infection status, dramatically reduced malaria parasitaemia, almost halved the rates of anaemia, and significantly improved cognitive ability [16]. In light of these promising results, it is important to replicate the results in other epidemiological settings. It is also clearly important to obtain information on the operational costs and cost-effectiveness of a delivery model for school-based IPT which can be implemented as part of an integrated school health programme. School health programmes already provide school children with deworming and micronutrients [17] and offer major cost advantages because of the use of the existing school infrastructure and the fact that the target population represents an accessible and relatively stable group. Evidence from existing programmes indicates that school-based delivery of anthelmintics is extremely cost-effective [18].

This paper aims to estimate the costs and cost-effectiveness of IPT delivered by teachers in Bondo District of Nyanza Province, western Kenya. Economic costs are calculated in order to help inform resource allocation and allow for comparison with alternative school-based intervention and across various health interventions, whereas financial costs are provided to inform replication of the approach. The analysis also explores how costs vary with the drug price and organization of delivery of the intervention as well as with differences in intervention effectiveness and background prevalence of anaemia.

## Methods

### Description of the IPT trial

A cluster-randomised, double-blind, placebo-controlled trial was conducted in 30 schools (15 intervention and 15 control schools) in Bondo District, western Kenya, where malaria transmission is stable and perennial [19]. The study design and results have been detailed elsewhere [16]. The main objective of the trial was to determine the efficacy of IPT given at four-monthly intervals (once a school term) against anaemia (defined as haemoglobin<110 g/L). The trial also evaluated the impact of intervention on the prevalence and intensity of *Plasmodium falciparum *infection, mean haemoglobin, sustained attention and educational achievement. Children, aged 5–18 years, received one dose of sulphadoxine-pyrimethamine (SP) and three daily doses of amodiaquine (AQ), on three occasions within a 12-month period (IPT was given once each school term). IPT was first given in May 2005, coinciding with a seasonal peak in transmission, and then repeated in September 2005 and January 2006. During the trial, treatment was provided by the research team during scheduled visits to schools, in close collaboration with district teams from the Ministry of Health (MoH) and Ministry of Education (MoE). Mass treatment with albendazole for soil-transmitted helminth infections was also provided to children in all 30 schools. Cross-sectional parasitological and haematological surveys were conducted in all schools in February 2005 and in March 2006, at the end of the intervention period.

### Implementation of teacher administrated programme of IPT

The present study estimates the costs of a school-based delivery of IPT in Bondo District, covering a school population in 2006 of approximately 74,000 school children. IPT comprises a single dose of SP on the first day and AQ as a daily dose over the course of three days. In order to reflect programmatic implementation it is assumed that IPT is delivered by teachers in schools, with supervision provided by staff of the District Health Office (DHO) at local levels and of the Division of Malaria Control (DoMC), MoH at a national level. Justification of such a delivery model is that it represents a well established delivery model of anthelmintics and micronutrients as part of school health programmes. Furthermore, previous studies demonstrate the ability of teachers to provide presumptive treatment for clinical malaria to schoolchildren in schools [13-15].

### Cost analysis

Cost data were collected from expenditure and salary records (budgetary records where expenditure data were not available) and from interviewing key district and national officials. Data collection was based on an itemized menu approach [20] and was undertaken between June–August 2006, shortly after the conclusion of the trial. Results are presented from a provider (national control programme) perspective, including those costs incurred by the MoE. Costs to households were excluded since costs of accessing treatment in their own schools were considered negligible. Both financial and economic costs were estimated. Financial costs represent monies paid for the implementation of the intervention by the programme at national and local levels. Economic costs include the opportunity cost of using existing government staff (including teachers) to implement the programme and these costs were estimated from government pay scales. Research costs were not included, but care was taken to include costs associated with programme monitoring. All costs were converted from Kenyan shillings to US dollars using the average exchange rate in 2006: 67.8 Kenyan Shillings = US$ 1 . Capital costs with a lifespan longer than 12 months were annuitized using a discount rate of 5% following standard practice [20].

Programme implementation costs were differentiated between initial set-up and recurrent costs, and separated into the following categories: 1) drug procurement; 2) drug distribution; 3) national supervision; 4) local supervision; 5) training; and 6) drug administration. Office equipment, training of trainers and community sensitization were the main set-up costs. The need for annual refresher training of teachers was assumed given high rates of staff turnover in schools, and included as a recurrent cost. Unit costs are provided in the supplementary information [Additional file 1].

Wholesale drug prices for preferred MoH brands were used. Drugs were stored at the district hospital, with relevant storage costs estimated. Ten percent drug wastage was assumed to account for expiration and loss of drugs. On the basis of discussions with local officials it was decided to model delivery costs on the basis of the distribution system used by the MoE to distribute anthelmintic tablets for deworming to schools. Subsequently, transport, personnel and supervision costs were estimated assuming that one vehicle can distribute drugs to ten schools per working day, with relevant costs for storage in the district included.

Programme supervision at the national level was estimated by assuming that a focal person at the DoMC would spend 10% of their time overseeing the programe, with relevant office costs included. These national supervision costs were estimated by modelling a national IPT programme in the 27 highly malaria endemic districts of Kenya and apportioning relevant costs to Bondo district. Local supervision of treatment was assumed to be logistically similar to supervision of school-based deworming, where health centre staff supervise treatment and deal with any side-effects arising, and costs were calculated accordingly.

Training was assumed to involve two components: first, the training of trainers and district officials (including 22 trainers and lasting three days); second, the training of teachers (two teachers from each school and lasting one day). Training of trainers is assumed at the beginning of the programme and thus represents set-up costs, whereas training of teachers is considered a recurrent cost. Training aimed to enhance awareness of malaria in schoolchildren and provide specific skills that would enable non-health staff to deliver IPT in schools. Health personnel were briefed on how to provide supervision and support to teachers. Training costs were based on previous experience in implementing school-based deworming and included opportunity costs of staff time valued at full salary costs (including tax and allowances), and costs related to transport, venue hire and supplies. Costs of community sensitization were estimated based on the time of two teachers per school taken to hold two parent teacher association meetings in schools (three hours per meeting) and included in the recurrent costs.

Administration of IPT included several activities with associated costs: photocopying of treatment instructions and report forms; teacher time spent on preparation, treatment and reporting. Retrospective estimates of time taken to deliver IPT during the efficacy trial were obtained through interviews with teachers in the intervention schools and used to calculate the opportunity cost of the time it would take teachers to administer IPT. It is assumed no payment would be made to teachers to deliver treatment. Additional supplies used during drug administration included treatment registers and glucose (to be co-administered with the bitter-tasting drugs to minimize non-compliance with treatment).

### Estimation of cost-effectiveness

Effectiveness was assessed in terms of cases of (i) anaemia averted and (ii) *P. falciparum *parasitaemia averted, and was derived from unadjusted results of the intervention trial, calculated on the basis of intention-to-treat [16]. Both costs and effects were estimated for the intervention compared to the counter-factual of "do-nothing". Outcome measures were cost per child treated per year, the cost per anaemia case averted and cost per case *P. falciparum *parasitaemia averted.

### Sensitivity analysis

A one-way sensitivity analysis was performed around both the main assumptions concerning costs and effectiveness. Specifically, as the price of different antimalarial drugs varies substantially and may also change over time and in different settings, drug prices were varied to 50% and 200% of current drug prices. Drug wastage was varied from 0% and 20%. An increase in personnel costs of 20% was also investigated. Finally, the discount rate (base case 5%) was varied to 3 and 7% and the exchange rate was varied to the highest and lowest levels reported for 2006 (1 US$ = Kenyan Shillings 74.5, 67.1).

As well as varying costs, differences in the effectiveness of IPT and the background level of anaemia were explored in the sensitivity analysis in order to explore potential cost-effectiveness under operational conditions and in different epidemiological settings. Specifically, the effectiveness of IPT in reducing anaemia was changed from 50% to 30% and 10%. The effectiveness in reducing malaria parasitaemia was changed from 88% to 60% and 40%. Finally, background prevalence of anaemia was varied from 12.6% to 30% and 50%.

For the purpose of estimating costs of IPT using alternative drug regimes, the choice of drugs was changed to include two relevant alternatives: 1) SP alone and 2) Dihydroartemisinin-Piperaquine (DP) – two drug regimes currently being investigated in a treatment efficacy trial against asymptomatic parasitaemic among schoolchildren in Uganda. In the Bondo intervention trial, IPT was given three times a year to correspond with the three school terms [16]; however because this could be reduced to twice a year by delivering IPT during the two main malaria seasons, the costs of biannual IPT delivery was also modelled. Note that only cost implications were assessed; effectiveness of alternative drug regimes remains unknown at present.

### Ethical clearance

Ethical clearance for the cost analysis, as well as the IPT trial, was given by the ethical committees of Kenyatta National Hospital, Kenya and London School of Hygiene & Tropical Medicine.

## Results

### Programme costs

In Bondo district, there were in 2006 approximately 74,000 schoolchildren attending 62 schools. The overall financial cost (i.e. market-value transaction costs) of a school-based, teacher administered IPT programme to all schools in the district was estimated to be US$ 88,859. The overall economic cost (i.e. financial plus opportunity costs of teachers and government officials' time) was estimated to be US$ 139,120. Table 1 presents the economic costs of the programme by major cost components. Set-up costs, namely equipment and initial training of trainers, accounted for 13.2% of overall costs whilst recurrent costs account for 86.8%. The estimated economic cost per child treated per year in Bondo district was US$ 1.88. The financial cost was US$ 1.20 per child treated per year. The set-up cost was estimated to be US$ 0.25 per child, and the annual recurrent cost US$ 1.63 per child treated per year.

**Table 1:** Annual economic cost of school-based IPT by major cost components (see additional file for breakdown of costs)

**Major cost components**	**Annual Cost (US$)**	**% of total**
**SET-UP COSTS**		
Equipment	3,543	2.6
Salaries (opportunity costs)	5,019	3.6
Per diems	5,275	3.8
Stationary	425	0.3
Fuel	133	0.1
Insurance/maintenance	25	0.0
Accommodation and food	3,846	2.8
**Total set-up costs**	**18,266**	**13.2**

**RECURRENT COSTS**		
Drugs	53,298	38.4
Stationary	2,498	1.8
Other consumables	2,044	1.5
**Total consumables**	**57,840**	**41.7**
		
Salaries (opportunity costs)	40,763	29.4
Incentives	1,217	0.9
Per diems	3,526	2.5
**Total Personnel**	**45,506**	**32.8**
		
Fuel	4,324	3.1
Insurance/maintenance	1,056	0.8
Local travel reimbursement	6,360	4.6
**Total Transport**	**11,740**	**8.5**
		
Accommodation and food	5,363	3.9
Other	124	0.1
		
**Total recurrent costs**	**120,573**	**86.8**

**Total**	**138,839**	**100**

The largest cost component was the purchase of SP and AQ, which contributed to 38.4% of overall costs. In terms of main activities, training of district staff and teachers was the most expensive activity (22.3%), followed by drug delivery costs (20.6%), transportation costs (10%) and local supervision costs (6.4%). The costs of community sensitization were minimal (2.3%) and the costs of national supervision were negligible (<0.01%).

### Effectiveness and cost effectiveness

In the unadjusted statistical analysis of data from the efficacy trial, delivery of IPT was associated with a 50% reduction in the proportion of children that are anaemic [16]. IPT was also estimated to reduce the prevalence of *P. falciparum *parasitaemia by 88%. On the basis of economic cost estimates, the cost per case of anaemia averted was found to be US$ 29.84. The cost per case of *P. falciparum *parasitaemia averted was US$ 5.36.

### Sensitivity analysis

The variables used in the sensitivity analysis and the effects on the overall costs and cost-effectiveness are shown in Table 2. If the price of SP decreased by 50%, the unit cost of treatment per child per year reduces from US$ 1.88 to US$ 1.61. If the cost of SP doubled, the unit cost increases to US$ 2.30. The corresponding figures for halving or doubling the price of AQ are US$ 1.55 and US$ 2.54. A 10% change in the wastage rate of drugs is associated with a 2.3% change in the price of IPT: if drug wastage decreased to 0%, the estimated cost decreases from US$ 1.88 to US$ 1.80 whereas at a wastage rate of 20%, costs would increase to US$ 1.95. Varying the exchange rate between the highest and the lowest rates reported for 2006 varies the price of IPT from US$ 1.88 to between US$ 1.78 (a decrease of 5%) and US$ 2.13 (an increase of 13.4%). Changing the discount rate had a negligible effect on the overall price of IPT. Increasing personnel costs by 20% increase overall costs to US$ 1.96.

**Table 2:** Sensitivity analysis variables and outcomes

**Variable**	**Baseline ** **value**	**Revised ** **values ** **in** **sensitivity ** **analysis**	**Justification**	**Impact on ** **cost per child** **per year** (Baseline: US$1.88)	**Impact on Cost effectiveness**	
					
					**Cost per case of anaemia averted** (Baseline: US$ 29.84)	**Cost per case of parasitaemia averted** (Baseline: US$ 5.36)
SP Price	US$ 0.08 per dose	200%	Drug price and brand are liable to change over time and across settings	US$ 1.61 US$ 2.30	US$ 25.56 US$ 36.51	US$ 4.59 US$ 6.55
AQ Price	US$ 0.12 per dose	50% 200%	As above	US$ 1.55 US$ 2.54	US$ 24.60 US$ 40.32	US$ 4.2 US$ 7.24
Drug Wastage	10%	0% 20%	Variations in expert opinion & literature	US$ 1.80 US$ 1.95	US$ 28.57 US$ 30.95	US$ 5.13 US$ 5.56
Exchange rate	US$ 1= Kes 67.81	US$ 1 = Kes 74.5 US$ 1 = Kes 67.1	Rate over 2006, and highest and lowest 2006 rates	US$ 1.78 US$ 2.13	US$ 28.25 US$ 33.81	US$ 5.07 US$ 6.07
Discount rate	5%	3% – 7%	5% is standard practice, with 3–7% used occasionally	Negligible change	Negligible change	Negligible change
Change in wages	2006	+ 20%	Salaries levels liable to change. 20% is a realistic change in next few years	US$ 1.96	US$ 31.11	US$ 5.58
Effectiveness: reduction in % anaemia	50%	10% 30%	Effectiveness may vary in different settings and when implemented outside of an efficacy trial	Not applicable	US$ 149.21 US$ 49.74	Not applicable
Effectiveness: reduction in % parasitaemia	88%	40% 60%	As above	Not applicable	Not applicable	US$ 11.84 US$ 7.89
Baseline % anaemia in Reduction in % parasitaemia	12.6%	30% 50%	Background nutritional levels vary between settings	Not applicable	US$ 12.53 US$ 7.52	Not applicable
Frequency of delivery	Three times a year	Twice a year	Yearly distribution as in efficacy trial versus seasonal distribution	US$ 1.42	Not applicable	Not applicable
Drug Choice	SP/AQ	SP alone DP	Current drug regime versus alternative drug regime currently under investigation	US$ 1.19. US$ 4.50	Not applicable	Not applicable

The results of the sensitivity analysis on the estimates of cost-effectiveness are also presented in Table 2. By changing costs, the cost per case of anaemia averted varied between US$ 24.60–40.32. The cost per case of malaria parasitaemia varied between US$ 4.20–7.24. Lower levels in the effectiveness of IPT were associated with markedly worst cost-effectiveness ratios (Table 2). Cost effectiveness depended most on the background prevalence of anaemia: in settings where 30% of children are anaemic the cost per case of anaemia averted is US$ 12.53 and where prevalence of anaemia is 50%, the cost per case of anaemia averted is US$ 7.52.

### Alternative IPT regimes

Because drug price is the primary cost driver, varying the type of drug used will considerably alter the overall cost of school-based delivery of IPT; effectiveness of alternative regimes is unknown at present. The cheapest IPT drug option would be to deliver SP alone: approximately US$ 1.19 per child per year (delivered three times a year), 33% cheaper than SP/AQ. Conversely delivering DP three times a year would cost US$ 4.50 per child which would be 139% more expensive than SP/AQ. For DP to be financially comparable to the current price of AQ/SP, its cost per dose would have to fall from approximately US$ 1 per treatment dose to US$ 0.20. Decreasing the frequency of delivery from three times to twice a year would decrease the cost of IPT from US$ 1.88 to US$ 1.42 (a 24% decrease). The effectiveness of alternative drug regimes and biannual treatment is unknown at present.

## Discussion

Numerous studies have investigated the cost-effectiveness of malaria control measures in Africa [21-25]. This study provides, to our knowledge, the first estimates of the cost and cost-effectiveness of a school-based malaria intervention and suggests that IPT administered by teachers is a potentially cost-effective school-based strategy, meriting further investigation.

Differences in outcome measures, target population, methodology and assumptions make it difficult to compare our results with other community-based malaria control interventions. Nonetheless, in terms of affordability, the per capita cost of IPT through schools (US$ 1.88 per child treated per year) compares favourably with estimated costs of other malaria interventions including insecticide treated nets (ITNs) and indoor residual spraying (IRS), which are considered 'attractive' low cost interventions for both low and middle income countries. ITNs are estimated to cost between US$1.40 and 3.85 per person protected per year [24] and IRS is estimated to cost between US$0.88 and 3.48 per person protected per year [26].

The costs of a school-based IPT programme are primarily driven by drug price and cost of personnel which account for 38.4% and 39.6% of the overall annual costs of treating a child, respectively. Any decrease in drug price over time will therefore act to increase the future financial attractiveness of IPT, 50% decrease in the price of AQ or SP would show a decrease in the price of IPT from US$ 1.88 to US$ 1.61 or US$ 1.55, respectively. The converse is true of personnel costs which are expected to rise over time and will decrease IPT's economic attractiveness: a 20% wage increase in Kenya would be associated with an increase in IPT price from US$ 1.88 to US$ 1.96 per child treated. This change in overall price is relatively low considering that personal costs are the largest cost driver and demonstrates the robustness of the price of IPT to short term changes in unit costs.

Set-up costs accounted for 13.2% of overall costs whilst recurrent costs account for 86.8%, equivalent to annual programme cost of US$ 1.63 per child treated per year. As an IPT programme is rolled-out, less frequent training of trainers would be required, further reducing personnel costs. Moreover, the simultaneous delivery by teachers of both IPT and deworming as part of an integrated school health package will yield what economists call economies of scope – the joint delivery of two health care interventions will be cheaper than a separate delivery of the same interventions – resulting in lower average delivery costs [27]. These factors would serve to reduce per capital costs. The costings presented here represent a maximal cost of delivering IPT, as no cost-sharing with other treatment programmes has been assumed in our estimates. The incremental cost-effectiveness of school-based IPT could therefore prove to be substantially lower than we have estimated.

In terms of cost-effectiveness, previous estimates for malaria control use a variety of outcome measures, including cost per death averted or cost per disability adjusted life year (DALY) averted. The DALY model of malaria is based the number of deaths and the number of years lived with disability from malaria episodes, severe anaemia and neurological sequelae, as defined in the Global Burden of Disease 2002 study [28]. The impact of IPT on these outcomes was not assessed and therefore a more intermediate health outcome of anaemia was selected since this was relatively common in the population, was a more epidemiologically relevant outcome in older children, and was able to be assessed empirically. The choice of anaemia also enables our results to be compared with other school-based interventions, notably school-based deworming. In Uganda, the cost effectiveness of school based anthelmintic treatment (using albendazole and praziquantel) ranged between US$ 1.70 and US$ 9.51 per anaemia case averted in different districts [29]. Similarly, a study in Tanzania reported the cost per case of anaemia averted to be US$ 7.43 [30]. Whereas the costs of anthelmintic drugs are very low (< $ 0.20 per child), anti-malarial drugs are more expensive, partly explaining the higher costs per anaemia case averted of school-based IPT (US$ 29.84) compared to school-based deworming (US$ 1.70–9.51). However, the cost of IPT per anaemia case averted will inevitably vary with the prevalence of anaemia, and so cost-effectiveness is likely to be most favourable where need is greatest: the cost per case of anaemia averted is US$ 12.53 in settings where prevalence of anaemia is 30% and US$ 7.52 where prevalence is 50%.

In addition to the health benefits of reducing anaemia in the intervention trial, there were immediate cognitive benefits [16]. Two tests were administed, both assessing sustained attention, the ability to concentrate on a task for an extended period of time. In the intervention group, scores for sustained attention were higher compared to the control group with an effect size of 0.18 in one test and 0.48 in the other. Cognitive abilities predict subsequent wages either directly [31] or through the long-term effects on increased educational attainment [32]. Through these mechanisms there may therefore also be education-related returns on the investment in IPT.

Affordability and cost-effectiveness are clearly important determinants of the long-term sustainability of the school-based IPT, but also crucial are feasibility and acceptability to the teachers, parents, health-workers and the wider community. Here, a key issue will be drug choice. Drugs best suited to mass treatment programmes should be cheap, easy to administer – preferably as a single dose – and well-tolerated with minimal side-effects. For intermittent preventive treatment of malaria, a long half-life is also advantageous. In this regard, SP alone and DP are both good candidates for school-based IPT that are being investigated in an ongoing trial in Uganda, although SP's future effectiveness may be limited by increasing parasite resistance against this drug. Alternative drug regimes will vary in price, dependant on both the cost per dose and the number of required doses. The present study showed that using SP alone would decrease the cost of treatment from US$ 1.88 to US$ 1.19 per child per year. Conversely, using DP would considerably increase the price of IPT from US$1.88 to US$ 4.50 per child per year. Although the effectiveness of these alternative IPT regimes has yet to be determined, it is apparent that the drugs chosen should be cheap, efficacious and simple to deliver in order to maximize the cost effectiveness of an IPT programme. In the efficacy trial, IPT was delivered three times per year. The current analysis shows that delivering IPT twice a year in association with the two main malaria peaks would decrease the price of IPT by approximately 24% (from US$ 1.88 to US$ 1.42 for SP/AQ).

A particular strength of this analysis is that costs are based on relevant information from the efficacy trial as well as the current structure of school deworming programmes, using best available data on relevant costs. However, the results are also subject to a number of assumptions including the discount rate, the drug wastage rate and the exchange rate. The sensitivity analysis showed, however, that the overall cost of IPT is relatively robust to these assumptions. In terms of extrapolation to other settings, Bondo District is representative of areas of stable perennial malaria transmission in East Africa. Similarly, the district's educational infrastructure is typical of rural East Africa. Factors which may vary in other settings include varying distances between schools and population densities which may alter the cost of IPT due to changing logistical needs. There may also be savings in terms of teacher training as the programme is rolled-out.

The primary limitation of the current analysis is that effectiveness is estimated from the efficacy trial where the study team delivered treatment. Programmatic delivery of IPT by teachers as part of a school health programme may, however, result in lower levels of effectiveness, which will in turn influence cost-effectiveness. The effectiveness of IPT may also vary according to the intensity and seasonality of malaria transmission. Although this aspect was explored through sensitivity analysis, in practice the effectiveness of school-based IPT is still largely unknown. To investigate some of these issues, parallel studies are currently planned in coastal Kenya and sahelian Senegal to assess the external validity of the study findings and to investigate further the long term educational gains of IPT in school.

IPT is one of a number of possible malaria control strategies which could be able to be delivered through schools [12]. The use of ITN has been associated with a 46% reduction in the prevalence of anaemia among Kenyan schoolgirls, aged 12–13 years [33], but was less effective in preventing anaemia among younger children. Another study among children in boarding schools in Kenya found that the use of bednets was associated with a decreased incidence of malaria [34]. Previously, global ITN guidelines focused primarily on providing ITNs for use by children under the age of 5 years and pregnant women. However, it has been recently recognized that protecting all community members yields enhanced health benefits and social equity, and WHO now recommends that Long-Lasting Insecticide Nets (LLINs) should be distributed freely or should be highly subsidized and used by all community members, including schoolchildren [35].

## Conclusion

The results of this analysis suggest that the per child cost of IPT in schools fall within the range of the per capita costs of other malaria control strategies. In terms of cost-effectiveness, IPT delivered by teachers is estimated to be less attractive in terms of per anaemia case averted than school-based deworming programmes. However, the cost-effectiveness of school-based IPT is likely to be highest where anaemia is widespread and need is, therefore, greatest. Furthermore, the simultaneous delivery by teachers of both IPT and deworming as part of an integrated school health package will yield further cost savings, so-called economies of scope. The relative effectiveness of IPT and deworming is currently unknown because the impact of IPT observed in the efficacy trial was on top of any effect of deworming since all children in the efficacy trial received anthelmintics. In addition to the efficacy of the intervention, cost-effectiveness will be influenced by the choice of drug and frequency of treatment. Further investigation of the effectiveness of school-based IPT in differing epidemiological and programmatic settings is clearly warranted.

## Competing interests

The authors declare that they have no competing interests.

## Authors' contributions

The cost-effectiveness study was conceived by SB with input from SEC, DHM, MCHJ and BBAE. MT collected the cost data, supported by JKN, WA and BBAE. MT, DHM and SB undertook the cost analysis. SB wrote the first draft of the paper and all authors contributed to, read and approved the final manuscript.

## Supplementary Material

Additional file 1 Unit costs of IPT delivered by teachers: resources, quantities and unit costs. Information on the resources employed, the quantities consumed and unit costs to deliver IPT through schools according to main activity is provided. Click here for file
