# Supplementary material for: Costs and cost-effectiveness of delivering intermittent preventive treatment through schools in western Kenya
Source: Malar J. 2008 Sep 30;7:196. doi: 10.1186/1475-2875-7-196 (PMC2564968; doi:10.1186/1475-2875-7-196)
Supplement: Additional file 1 — Unit costs of IPT delivered by teachers: resources, quantities and unit costs. Information on the resources employed, the quantities consumed and unit costs to deliver IPT through schools according to main activity is provided. [file 1475-2875-7-196-S1.doc]

**Supplementary Information. Temperley et al.**

**Unit costs of IPT delivered by teachers: resources, quantities and unit costs**

The resources employed, the quantities consumed and unit costs are described below under the different activities.

| **Teacher Training** | **Unit** | **Kes** | **US$** |
| --- | --- | --- | --- |
| District health officials salaries | per month | 44,000 | 649.0 |
| District health officials per diems | per day | 1,000 | 14.7 |
| District Education officials salary | per month | 42,000 | 619.5 |
| DoE incentives | per day | 1,000 | 14.7 |
| TAC personnel salaries | per month | 17,000 | 250.7 |
| TAC per diems | per day | 500 | 7.4 |
| ECD personnel salary | per month | 23,000 | 339.2 |
| ECD per diems | per day | 500 | 7.4 |
| Teachers salary | per month | 16,113 | 237.7 |
| Posters | per poster | 10 | 0.1 |
| Leaflets | per leaflet | 3 | 0.0 |
| Flip charts | per chart | 2,000 | 29.5 |
| Pens | per pen | 10 | 0.1 |
| Pads | per pad | 50 | 0.7 |
| Folder | per folder | 40 | 0.6 |
| Name tags | per tag | 30 | 0.4 |
| Felt pen pack | per pack | 1,000 | 14.7 |
| Masking tape | per roll | 100 | 1.5 |
| Stapler and staples | per stapler and staples | 250 | 3.7 |
| Food and venue hire in Bondo | per person per day | 640 | 9.4 |
| Teachers travel allowance | per day | 500 | 7.4 |
| **Training of Trainers** |  |  |  |
| Facilitators salary | per month | 70,000 | 1032.4 |
| District health officials salaries | per day | 2,200 | 32.4 |
| District Education officials salary | per day | 2,100 | 31.0 |
| TAC personnel salaries | per day | 850 | 12.5 |
| ECD personnel salary | per day | 1,150 | 17.0 |
| Facilitators per diem | per day | 4,000 | 59.0 |
| District health officials per diem | per day | 3,000 | 44.2 |
| District Education officials per diem | per day | 2,500 | 36.9 |
| TAC personnel per diem | per day | 500 | 7.4 |
| ECD personnel per diem | per day | 500 | 7.4 |
| Posters | per poster | 10 | 0.1 |
| Leaflets | per leaflet | 3 | 0.0 |
| Flip charts | per chart | 2,000 | 29.5 |
| Pens | per pen | 10 | 0.1 |
| Pads | per pad | 50 | 0.7 |
| Folder | per folder | 40 | 0.6 |
| Name tags | per tag | 30 | 0.4 |
| Felt pen pack | per pack | 1,000 | 14.7 |
| Masking tape | per roll | 100 | 1.5 |
| Stapler and staples | per stapler and staples | 250 | 3.7 |
| Venue hire food and accommodation Kisumu | per day per person | 2,000 | 29.5 |
| District officials travel allowance | per training session | 500 | 7.4 |
| Vehicle purchase cost (facilitator travel) | per vehicle | 2,758,000 | 40678.5 |
| Vehicle maintenance (facilitator travel) | per year | 12,0000 | 1769.9 |
| Vehicle insurance (facilitator travel) | per year | 95,000 | 1401.2 |
| National drivers salary (Facilitator travel) | per day | 2,100 | 31.0 |
| National drivers per diem (Facilitator travel) | per day | 1,500 | 22.1 |
| **Sensitization** |  |  |  |
| Teachers salary | per day | 805 | 11.9 |
| **Transport** |  |  |  |
| Vehicle purchase | per vehicle | 2,758,000 | 40678.5 |
| Vehicle maintenance | per year | 120,000 | 1769.9 |
| Vehicle insurance | per year | 95,000 | 1401.2 |
| Local education officer salary | per day | 2,200 | 32.4 |
| Local education officer per diem | per day | 3,000 | 44.2 |
| Local driver wage | per day | 650 | 9.6 |
| Local driver per diem | per day | 1,800 | 26.5 |
| National driver salary | per day | 2,100 | 31.0 |
| National driver per diem | per day | 1,500 | 22.1 |
| **Supervision** |  |  |  |
| Head DoMC | per day | 7,500 | 110.6 |
| Focus person DoMC | per day | 3,500 | 51.6 |
| **Office costs** |  |  |  |
| Telephone with two handsets and single ext line | per year | 3,231 | 47.7 |
| Personal comp (inc scanner and power backup) | per year | 25,851 | 381.3 |
| Fax | per year | 10,340 | 152.5 |
| Laser printer | per year | 8,918 | 131.5 |
| Accounting software (inc training) | per year | 3,619 | 53.4 |
| Software costs (MS office) | per year | 1,938 | 28.6 |
| Rent including taxes and tariffs | per year | 141,024 | 2080.0 |
| Utilities(water, electric, telephone, security, parking) | per year | 100,731 | 1485.7 |
| Cleaning and messenger services | per year | 40,292 | 594.3 |
| Internet and IT support | per year | 40,292 | 594.3 |
| Accounting | per year | 80,585 | 1188.6 |
| Mail and courier | per year | 40,292 | 594.3 |
| Transport (taxi) | per year | 134,309 | 1981.0 |
| **Treatment** |  |  |  |
| Register | per book | 200 | 2.9 |
| Glucose | per 500 grams | 60 | 0.9 |
| Amodiaquine | per dose | 8.7 | 0.1 |
| SP | per dose | 6.1 | 0.1 |
| Drug wastage | Percent | 20 | 0.3 |
| Store room space | per year per district | 2,231 | 32.9 |
